# Induction of the Nrf2 Pathway by Sulforaphane Is Neuroprotective in a Rat Temporal Lobe Epilepsy Model

**DOI:** 10.3390/antiox10111702

**Published:** 2021-10-27

**Authors:** Sereen Sandouka, Tawfeeq Shekh-Ahmad

**Affiliations:** Institute for Drug Research, The School of Pharmacy, Faculty of Medicine, The Hebrew University of Jerusalem, Jerusalem 91120, Israel; Sereen.Sandouka@mail.huji.ac.il

**Keywords:** epilepsy, epileptogenesis, Nrf2-KEAP1 pathway, oxidative stress, SFN

## Abstract

Epilepsy is a chronic disease of the brain that affects over 65 million people worldwide. Acquired epilepsy is initiated by neurological insults, such as status epilepticus, which can result in the generation of ROS and induction of oxidative stress. Suppressing oxidative stress by upregulation of the transcription factor, nuclear factor erythroid 2-related factor 2 (Nrf2) has been shown to be an effective strategy to increase endogenous antioxidant defences, including in brain diseases, and can ameliorate neuronal damage and seizure occurrence in epilepsy. Here, we aim to test the neuroprotective potential of a naturally occurring Nrf2 activator sulforaphane, in in vitro epileptiform activity model and a temporal lobe epilepsy rat model. Sulforaphane significantly decreased ROS generation during epileptiform activity, restored glutathione levels, and prevented seizure-like activity-induced neuronal cell death. When given to rats after 2 h of kainic acid-induced status epilepticus, sulforaphane significantly increased the expression of Nrf2 and related antioxidant genes, improved oxidative stress markers, and increased the total antioxidant capacity in both the plasma and hippocampus. In addition, sulforaphane significantly decreased status epilepticus-induced neuronal cell death. Our results demonstrate that Nrf2 activation following an insult to the brain exerts a neuroprotective effect by reducing neuronal death, increasing the antioxidant capacity, and thus may also modify epilepsy development.

## 1. Introduction

Epilepsy is a chronic disease of the brain that affects over 65 million people worldwide [1], representing 1–2% of the population, making it one of the most common neurological diseases globally. It is common in all age groups and is associated with a burden of socio-economical, behavioural, psychiatric, and other medical issues for patients, their caregivers, and society [2]. Currently, there are over 27 FDA approved anti-epileptic drugs (AEDs). Even though these AEDs medications are used to treat the symptoms of the disease (i.e., control the seizures), the disease is not adequately controlled in a third of epileptic patients [3], and the core of the illness remains intact and perplexing dilemma.

Acquired epilepsy, which constitutes up to 50% of all epilepsy cases, is initiated by neurological insults, such as stroke, traumatic brain injury (TBI), and status epilepticus (SE) [4]. The initial insult is followed by a latency period referred to as epileptogenesis, a progressive activity initiated in the brain following insults or injury, which results into a dynamic sequence of events, including numerous structural, cellular, and molecular changes occurring over time, with a reactive response that generates abnormal patterns of electrical discharges in discrete brain regions resulting in the eventual development of epilepsy, where the brain is capable of generating spontaneous seizures [5,6].

The generation of reactive oxygen species (ROS) and the induction of oxidative stress (OS) are common sequelae of brain-damaging insults [7]; they are considered cytotoxic mechanisms that play a fundamental role in the pathophysiology of epilepsy and contribute to neuronal cell death [8]. OS occurs as a consequence of diminished endogenous antioxidants and repair capacity that cannot encompass the normal oxidant burden or as a consequence of an increased oxidant burden, which overwhelms the endogenous antioxidants and repair capacity [9].

In recent years, there is growing evidence that inhibiting ROS generation can ameliorate neuronal damage in seizures and epilepsy [10,11,12]. One promising antioxidant strategy that has been studied in neurological disorders, including epilepsy, is the upregulation of the transcription factor, nuclear factor erythroid 2-related factor 2 (Nrf2), a major transactivator of cytoprotective genes, such as NAD(P)H: quinone oxidoreductase 1 (NQO1) and heme oxygenase 1 (HO-1) [13]. Upon OS or exposure to electrophiles, the cytoplasmic regulator of Nrf2, Kelch-like ECH associated protein 1 (Keap 1), is inactivated due to electrophile binding, leading to dislodging of Nrf2 and allowing it to escape proteasomal degradation, thus, translocating into the nucleus, where it activates target detoxifying and antioxidant genes [14].

Indeed, several studies have recently shown the protective effect of Nrf2 activation in many pathologies [15], including in epilepsy. Nrf2 and Nrf2-dependent gene products as well as OS markers were significantly increased after seizure in the hippocampus of amygdala kindling rats [16]. Following a status epilepticus (SE) in mice, Nrf2 overexpression significantly decreased the generalized seizures with a profound reduction in microglial activation [17]. We recently demonstrated that activation of Nrf2 through the inhibition of keap1 using cyanoenone triterpenoid, omaveloxolone, can suppress the development of epilepsy in rat temporal lobe epilepsy (TLE) models alone or in combination with NADPH oxidase inhibitor, AEBSF [7,18,19].

Furthermore, we also reported that pharmacological Nrf2 activation via the naturally occurring Nrf2 activator sulforaphane (SFN), administered with the antioxidant N-acetylcysteine (NAC), has as a disease-modifying effect in rats subjected to electrically induced-SE [19]. SFN, a naturally occurring Nrf2 activator, an isothiocyanate compound found in cruciferous plants, such as cabbage and cauliflower, has been proposed as a possible treatment in several neurological diseases, including epilepsy [19]. However, SFN is non-selective, affecting many off-target proteins [20], which can explain why it exhibits proconvulsant activity [21]. Nevertheless, several studies have shown that SFN exerts antioxidant and protective effects in epileptic seizure models in mice and rats [22,23,24].

However, the neuroprotective effect as well as the antioxidant potential of SFN by its own had not yet been tested in an SE rodent model. Thus, our current study aims at investigating the antioxidant properties and the neuroprotective effects of SFN when given as monotherapy following kainic acid-induced-SE (TLE) model in rats.

Here, we show that activation of Nrf2 with SFN inhibits ROS production, increased the glutathione pool, and neuroprotects in an in vitro seizure-like activity model in primary cortical cultures. When administered to rats following KA-induced SE, SFN increased the levels of reduced GSH, decreased the levels of oxidized GSH, increased the total antioxidant capacity in the plasma and hippocampus, and decreased the neuronal cell death in the CA1 and CA3 regions of the hippocampus. Our results extend and confirm the antioxidant neuroprotective properties of SFN in a TLE rat model.

## 2. Materials and Methods

### 2.1. In Vitro Cortical Cell Cultures

Mixed cortical neurons and glial cells cultures were prepared from postnatal (P0-P1) Sprague–Dawley rat pups (Hebrew University breeding colony, Jerusalem, Israel) [7,11,25]. The pups were sacrificed, and their brains were quickly removed; neocortical tissue was isolated and immediately submerged in ice-cold HBSS (Ca^2+^, Mg^2+^-free, Sigma, H9394). The tissue was treated with 1% trypsin (Invitrogen) for 6–7 min at 37 °C, the trypsinization was followed by blocking by HBSS with FBS 20% (Foetal bovine serum, Invitrogen) and trituration to dissociate cells.

After counting the cells, the final neuronal cell suspension was plated on 25 mm round coverslips coated with poly-L-lysine (1 mg/mL, Sigma), and cultured in Neurobasal^®^ A medium supplemented with B-27 (Invitrogen) and 2 mM L-glutamine. Neocortical cultures were fed one to two times a week and maintained in a humidified atmosphere of 5% CO2 and 95% air at 37 °C in a tissue culture incubator. Experiments were carried out at 13–17 days in vitro (DIV) to allow for full maturation of the synapses of cells. Neurons were distinguished from glia for their distinct shape using phase-contrast imaging.

### 2.2. In Vitro Recording Solutions

Experiments were performed using HEPES buffered salt solution including artificial CSF or by omitting MgCl_2_ (low-Mg^2+^) (to induce the seizure model) at room temperature. (aCSF) composition (in mM): 125 NaCl, 2.5 KCl, 2 MgCl_2_, 1.25 KH_2_PO_4_, 2 CaCl_2_, 30 glucose and 25 HEPES, and pH adjusted to 7.4 with NaOH.

### 2.3. Measurements Rates of ROS Generation

To assess the rates of ROS production in the cytosol, dihydroethidium (DHE) (5 µM) was present in all solutions throughout the experiments without pre-incubation in order to avoid the accumulation of oxidized products. DHE is oxidized to the highly specific ROS indicator 2-hydroxyethidium (2HE). Experiments were conducted using three separate cultures and repeated on six coverslips. DHE was excited by illumination at 530 nm. We chose to perform measurements of ROS production rates with DHE at a single wavelength to detect only the 2HE. The rates of ROS increase were calculated at different time points (5, 10, and 15 min) after exposure to low-Mg^2+^ and were compared with rates recorded during a 1–3 min aCSF exposure period referred to as the baseline.

### 2.4. Imaging of Glutathione (GSH) Levels

To measure the GSH levels, the cell cultures were incubated with Monochlorobimane (MCB) (Sigma-Aldrich) was prepared as a 50 mM stock solution in DMSO and used at a final concentration of 50 μM for 1 h at room temperature. Either aCSF or low-Mg^2+^ aCSF solutions were used throughout the experiments. The cells were then washed, and images of the fluorescence of the MCB-GSH adduct were acquired using a cooled SCMOS imaging system. Experiments were repeated on six coverslips using at least three separate cultures.

### 2.5. Assessment of Neuronal Cell Death in Co-Culture

Neuronal cell death was determined following incubation with low-Mg^2+^ for 2 h at 37 °C. Cells were co-stained with propidium iodide (20 μM) (which stains nuclei with red fluorescence) and Hoechst 33342 (4.5 μM) (Sigma) (which is a cell membrane permeant, minor groove binding DNA stain) in a fluorescent live/dead assay. Experiments were conducted on six to seven coverslips for each treatment using at least three cultures. In each treated culture coverslip, five random fields were counted.

### 2.6. Live Cell Imaging

Fluorescence images were obtained on a Nikon Eclipse CiL (Nikon Instruments, Melville, NY, USA) epifluorescence inverted microscope with a 20× fluorite objective. Excitation light provided by a xenon arc lamp, the beam passing monochromator at 380, 490 or 530. Emitted fluorescence was detected by a cooled Zyla 4.2 sCMOS camera (Andor, Belfast, UK).

### 2.7. Real-Time RT-PCR Expression Analysis of Nrf2 and Related Genes In Vivo

The mRNA levels of Nrf2 (Accession number: NM_031789), NQO1(Accession number: NM_017000), and HO-1(Accession number: NM_012580) were determined by quantitative real-time PCR. The total RNA was extracted from 70 mg of rat cortex and 70 mg hippocampus tissue using TRI Reagent (Sigma-Aldrich, St. Louis, MO, USA). It was then quantified, and the purity was assessed by Nanodrop (Nanodrop Technologies, Thermo, Waltham, MA, USA) spectrophotometry; the 260:280 ratios were 1.8–2.0. Complementary DNA (cDNA) was synthesized from 1 μg of total RNA using GoScript™ Reverse Transcription System (Promega, Madison, WI, USA) with an oligo-dT_15_ primer.

The expression of the three selected redox-relevant genes was analysed by real-time PCR, which was performed with (CFX-bio Rad) instrument using SYBR Green (PerfeCTa SYBR Green FastMix, Quantabio). Reaction mixtures 15 μL of (7.5 μL of SYBR Green, 3 μL of primers (500 nM each), 1.5 μL of DEPC water and 3 μL of cDNA template was added to the mixture) were incubated for 10 min at 95 °C, followed by 40 cycles of denaturation at 95 °C for 5 s, and at 60 °C for 15 s. Finally, 5 s at 65 °C, and 30 s at 95 °C. For each mRNA, gene expression was corrected by the RNA GAPDH (Accession number: NM_017008) subunit content in each sample. The results were expressed with respect to the control group, which was normalized to 1, and the Pfaffl (2001) [26] method was used to calculate the relative expression of each gene. In all cases, each PCR was performed with duplicate samples.

The primers used were designed with the Primer Express Software 3.0 (Applied Biosystems), by using the Gen-Bank sequences as listed in Table 1. Primers were designed for Nrf2, NQO1, HO-1, and GAPDH. The specificity of the primers was tested by performing a BLAST search against the genomic NCBI database.

### 2.8. Western Blot Analysis of Nrf2 and NQO1

Rats were deeply anaesthetized using ketamine (100 mg/kg) and xylazine (10 mg/kg), then their brains were rapidly removed, and the cortices and hippocampi were dissected out, and washed with cold PBS. Rat cortex and hippocampus tissue (70 mg each) were homogenized and lysed with RIPA buffer (Thermo Scientific) with a protease inhibitor cocktail, and then protein concentrations were measured using BCA protein assay kit (Bio-rad). Equal amounts of protein (25 µg) were loaded onto 10% SDS-PAGE and proteins were transferred to a nitrocellulose membrane (Bio-rad, Hercules, CA, USA).

The membranes were then blocked with 5% non-fat milk for Nrf2, NQO1, and 5% BSA for β actin, in Tris-buffered saline for 2 h at room temperature. The membranes were then incubated overnight at 4 °C with primary antibodies against Nrf2 (ab31163, 1:1000), NQO1 (ab34173, 1:1000), and β actin (ab8226, 1:2000). The preparative membranes were reacted with appropriate secondary antibodies conjugated to HRP. The immunological complexes were visualized with electrochemiluminescence (ECL, Bio-rad). Band intensities were analysed by (Image lab, Bio-rad, Hercules, CA, USA) software.

### 2.9. Measurements of Reduced (GSH), Oxidized (GSSG) Glutathione Levels, and the (GSSG/GSH) Ratio

After tissue homogenization, protein concentrations were measured using BCA protein assay kit (Bio-rad), and the glutathione (GSH), glutathione disulfide (GSSG), and the GSH/GSSG ratio were measured using the GSH/GSSG Ratio Detection Assay Kit II (Fluorometric–Green; ab205811; Abcam) according to the manufacturer’s instructions.

### 2.10. Total Antioxidant Capacity (TAC)

The total antioxidant capacity (TAC) was determined using a commercially available assay kit (Abcam ab65329), which utilizes the conversion of Cu2^+^ ions to Cu^+^ through endogenous protein and small-molecule antioxidants, standardized to Trolox equivalents. Brain tissue (100 mg of cortex; 50 mg of the hippocampus) was homogenized in cold PBS and centrifuged at 4 °C for 10 min at 14,000 rpm, and supernatant was used for the analysis of TAC. Plasma was directly used for the assay. Samples were diluted (1:20) in sterile deionized water (DDW), and 10 μL of samples and 100 μL of Trolox standard were added to each well of a 96-well plate. The reaction was initiated by adding 100 μL of Cu2^+^ working solution to each well, and mixing the plate on an orbital shaker for 90 min at room temperature shielded from light. The colorimetric activity was measured by optical density (OD) at 570 nm, and the antioxidant capacity was calculated against the linear Trolox standard calibration curve (1–20 μM Trolox).

### 2.11. Assessment of Neuronal Cell Death In Vivo

The immunohistochemistry experiment was performed for the visualization of neurodegeneration and to characterize the neuronal damage using cell-specific markers. One week following induction of SE, rats were euthanized (using ketamine-100 mg/kg and xylazine-10 mg/kg) then perfused transcardially with 150 mL of cooled heparinized PBS followed by 4% paraformaldehyde (PFA) in PBS (Santa Cruz Biotechnology, Dallas, TX, USA). Brains were removed and left in 4% PFA/PBS overnight at 4 °C, and then cryoprotected with a 15–30% sucrose gradient over 3–4 days until the tissues sank.

### 2.12. Microscopy

After cryoprotection, brains were embedded in O.C.T. Compound (Scigen) and stored at −80 °C. For each animal, coronal sections (20 µm) selected from the hippocampus were cut in a cryostat (Leica CM1950) at −20 °C and fixed on poly-L-lysine coated slides (Thermo Fisher Scientific), then left to air dry at room temperature for 2 h. Brain sections were circled with a water repellent pen (Dako pen; Agilent), permeabilized with PBS, 0.2% Triton X-100 (Sigma) for 30 min, blocked with 4% goat serum (Sigma) for 2–3 h then washed three times for 10 min each with PBS. Sections were incubated overnight at 4 °C with a rabbit primary antibody against NeuN (1:500, ab177487, Abcam, Cambridge, UK) in a solution of PBS, 0.1% triton X-100 and BSA 1%.

Following three washes with PBS (10 min each); the sections were incubated with Alexa Fluor^®^ 488 goat anti-rabbit secondary antibody (1:500; ab150081, Abcam, Cambridge, UK) for 2 h at room temperature and kept in dark. The sections were washed three times with PBS (10 min each) and mounted with Vectashield and 4′,6-diamidino-2-phenylindole (DAPI) mounting medium (Vector Labs, Burlingame, CA, USA). Images were obtained at a resolution of 1024 × 1024 on Nikon confocal A1R microscope using a 20X objective.

Images were acquired at 405 nm excitation wavelength and 455 nm emission wavelength for DAPI, and excitation of 488 nm and emission of 520 nm for NeuN. Image analysis was performed using ImageJ software in a manual cell counting image-based tool, and investigators were blinded to treatment. Cell densities for individual animals represent the average densities of the particular region for 2-brain sections/rat in two to three images of ROI’s of CA1 and CA3 subfields of the hippocampus. The results are expressed as neurons per mm^2^.

### 2.13. Experimental Epilepsy Model

#### 2.13.1. Animals

Animal experiments were conducted in accordance with the Association for Assessment and Accreditation of Laboratory Animal Care International and approved by the Institutional Animal Care and Use Committee of the Hebrew University of Jerusalem (Approve number: MD-20-16254-5).

Male and Female Sprague–Dawley rats (180–220 g), a strain of the Hebrew University (obtained from Harlan, Jerusalem, Israel) were housed in groups with food and water available and *ad libitum*. Rats were kept under controlled environmental conditions (23 ± 1 °C; 50–60% humidity; 12-h light/dark cycle).

#### 2.13.2. KA-Induced SE Epilepsy Model

The well-validated kainic acid-induced status epilepticus (KA-SE) of the temporal lobe epilepsy (TLE) model was used to induce epilepsy in SD rats. Rats were injected intraperitoneally with Kainic Acid (Hello Bio, Bristol, UK) at a dose of 5 mg/kg hourly and continuously monitored for convulsive motor seizures until class III, IV, or V seizures were evoked (scored according to a modified Racine’s scale [27,28]). Once an animal began showing excessive inactivity or excessive activity (i.e., exaggerated running or jumping), subsequent injections were delayed or reduced to 2.5 mg/kg to avoid excessive toxicity and mortality. The endpoint for KA treatment was considered either when animals reached class V seizure (i.e., excessive rearing with concomitant forelimb clonus and falling) or when the total dose of KA reach 45 mg/kg. There was no difference in the weight of animals randomized to vehicle or SFN treatment groups (201 g ± 15 and 212 g ± 18, respectively).

#### 2.13.3. Drug Administration

Rats subjected to SE were randomized to treatment with either vehicle (20% polyethylene glycol (PEG) 300, 80% methylcellulose (MC, 0.5%) or SFN (Carbosynth, Ltd., San Diego, CA, USA, Cas No. 4478-93-7) dissolved in the same vehicle at a dose of 5 mg/kg once daily for 5 days. Control animals were treated with an equivalent volume and number of injections of vehicle as the SFN treated animals. At the end of SE, rats were given a subcutaneous (S.C.) injection of Glucose 5% (3–5 mL) and softened rat chow to minimize animal discomfort. The animals were sacrificed one week after and were used for either immunohistochemical or biochemical analysis.

#### 2.13.4. Statistical Analysis

Data are expressed as the mean ± standard error of the mean (SEM). The statistical analyses were performed using GraphPad Prism 8.4.3 software. Data were analysed using ordinary or repeated one-way analysis of variance (ANOVA) followed by Tukey’s multiple comparisons test. Values of *p* < 0.05 and *p* < 0.01 were considered statistically significant.

Sample sizes were chosen based on our previous experiences in the calculation of experimental variability. The numbers of animals used are described in the corresponding figure legends.

## 3. Results

### 3.1. SFN Decreases Rate of ROS Production, Increases Levels of Glutathione, and Decreases Epileptiform Activity-Induced Neuronal Death

**Figure 1:**
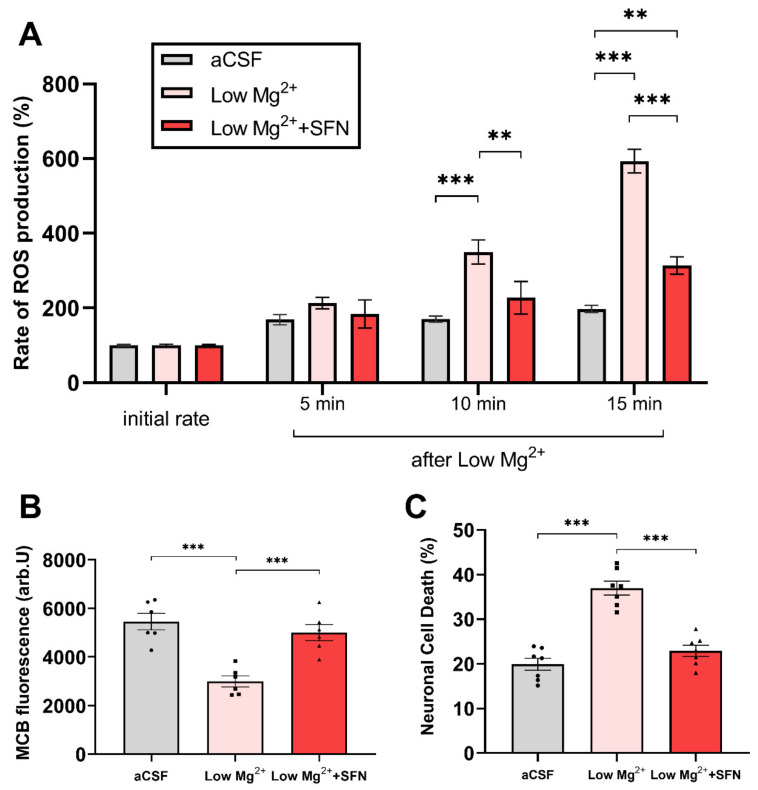
**SFN decreases the rate of ROS production, increases levels of glutathione, and decreases epileptiform activity-induced neuronal death.** (**A**). Bar chart summarizing the rates of ROS production (mean ± SEM) at 5, 10, and 15 min of neuronal cultures in artificial CSF (aCSF; *n* = 322 cells, six experiments), after the omission of Mg^2+^ from the extracellular solution (low Mg^2+^; *n* = 346 cells, six experiments), and cultures treated with SFN (5 µM, pre-treatment 24 hr) in low-Mg^2+^ condition (*n*= 358 cells, six experiments). Data were analysed by repeated measures one-way ANOVA [F (2, 12) = 46.63, *p* < 0.001, followed by Tukey’s multiple comparisons post hoc test. ** *p* < 0.01, *** *p* < 0.001 relative to the low-Mg^2+^ condition. (**B**,**C**). Levels of glutathione measured using MCB fluorescence (**B**), *n* = 6 experiments in all three conditions) and percentage of dead neurons (**C**), *n* = 7 experiments in all three conditions) in same conditions as in A: aCSF, low Mg^2+^, and low Mg^2+^ with SFN (5 µM, pre-treatment, 24 h). Data were analysed by one-way ANOVA (**B**): F (2, 15) = 18.62, *p* < 0.001; (**C**): F (2,18) = 43.21, *p* < 0.001] followed by Tukey’s multiple comparisons test. Data are expressed as the mean± SEM. ** *p* <0.01 and *** *p* <0.001, relative to the low-Mg^2+^ condition).

We first wanted to evaluate the effect of SFN on ROS generation in the in vitro epileptiform activity model, the low Mg^2+^ model (Figure S1). In keeping with previous studies, we found that induction of seizure-like activity by omitting magnesium from the medium induced 350% and 600% increase in the rate of ROS production, 10 and 15 min after exposure to low Mg^2+^, respectively (Figure 1A). SFN (5 µM, pre-treatment for 24 h) significantly reduced the rate of ROS generation during epileptiform activity 10 and 15 min after exposure to low Mg^2+^ (from 350% to 227%, and from 593% to 314% at 10 and 15 min, respectively; F (2, 60) = 46.63, *p* < 0.0001, Figure 1A).

Since epileptiform activity significantly decreases the levels of glutathione (GSH) indicating oxidative stress, we asked if pre-treatment of co-cultures with SFN can restore the GSH pool following 2 h of exposure to low Mg^2+^ induced epileptiform activity. Co-cultures exposed to low Mg^2+^ show an almost 50% decrease in GSH levels, which was almost completely restored by SFN (5 µM, pre-treatment for 24 h); (F (2, 15) = 18.62, *p* < 0.0001, Figure 1B and Figure S3).

Subsequently, we asked if the antioxidant effect of SFN can translate into a neuroprotective effect. Exposure of co-culture to low Mg^2+^ medium for 2 h resulted in a two-fold increase in neuronal cell death compared to aCSF media (20% and 38%, respectively, Figure 1C). Incubation of co-culture with 5 µM of SFN for 24 h significantly decreased the seizure-like activity-induced neuronal cell death (23%, F (2, 18) = 43.2, *p* < 0.0001, Figure 1C and Figure S2).

### 3.2. SFN Increases the Expression of Nrf2 and its Related Genes NQO1 and HO-1 Following Status Epilepticus In Vivo

We next sought to confirm the in vitro antioxidant effects of SFN following the kainic acid (KA)-induced status epilepticus (SE) temporal lobe epilepsy (TLE) model in rats. For this, we investigated the effect of SFN on the mRNA and protein expression of Nrf2 and Nrf2-associated genes (i.e., NQO1 and HO-1) in both cortex and hippocampus following induction of SE. Two hours after SE, rats were randomized to either SFN (5 mg/kg once a day for 5 days, with the first dose within 10 min following SE) or vehicle (equivalent volume and number of injections).

**Figure 2:**
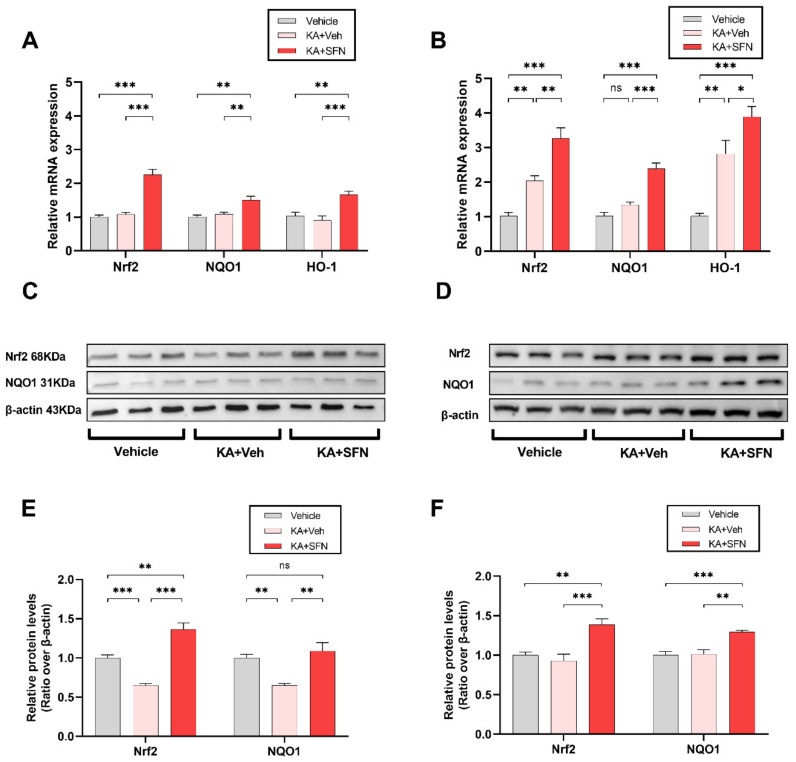
**SFN increases the expression of Nrf2, NQO1 and HO1 following in vivo SE.** Expression of Nrf2, NQO1, and HO1 mRNAs in cortex (**A**) and hippocampus (**B**), 7 days following vehicle treatment (no KA-SE, *n* = 6) and rats subjected to 2 h of KA-SE and treated either with SFN (5 mg/kg once a day for 5 days, first dose within 10 min following SE, *n* = 6) or vehicle (equivalent volume and number of injections, *n* = 6). (**C**,**D**). Representative immunoblots of Nrf2, NQO1, and of same animals as in (**A**) and (**B**) as detected by western blotting. E-F. Relative expression (over β-actin) of Nrf2 and NQO1 total proteins in cortex (**E**) and hippocampus (**F**) of blots in (**C**,**D**), respectively. Data are displayed as the mean ±SEM, * *p* < 0.05, ** *p* < 0.01, *** *p* < 0.001 analysed by one-way ANOVA followed by Tukey’s multiple comparisons test.

In the cortex, the results demonstrated that treatment with SFN was associated with significant increases in the mRNA expression levels of Nrf2, NQO1 and HO-1 by 2.1-, 1.4-and 1.8-fold, respectively, compared with the vehicle treated group (Figure 2A, Nrf2: F (2, 15) = 46.57, *p* < 0.0001; NQO1: F (2, 15) = 10.79, *p* = 0.0012; HO1: F (2, 15) = 12.78, *p* = 0.0006). A similar pattern of increase in the mRNA expression levels of all genes was also observed in the hippocampus (Figure 2B, Nrf2: F (2, 15) = 30.61, *p* < 0.0001; NQO1: F (2, 15) = 37.79, *p* < 0.0001; HO1: F (2, 15) = 26.06, *p* < 0.0001).

Furthermore, western blot analysis demonstrated that 1 week following 2 h of KA-SE, there was a significant decrease in total protein levels of Nrf2, and this was solely mediated gene NQO1 in the cortex when compared to vehicle treated rats [Figure 2C,E; Nrf2: F (2, 15) = 46.67, *p* < 0.0001, vehicle vs. KA + Veh: *p*= 0.0007; NQO1: F (2, 15) = 11.26, *p* = 0.0010, vehicle vs. KA + Veh: *p* = 0.0072]. Interestingly, no difference in the total protein levels of both Nrf2 and NQO1 was detected in the hippocampus [Figure 2D,F; F (2, 15) = 12.96, *p* = 0.0005, vehicle vs. KA + Veh: *p*= 0.7351; NQO1: F (2, 15) = 13.85, *p* = 0.0004, vehicle vs. KA + Veh: *p* = 0.9814].

In rats treated with SFN following KA-SE, the total protein levels of Nrf2 and NQO1 were markedly elevated compared with vehicle treated animals (whether subjected or not to KA-SE) in both the cortex and the hippocampus (Figure 2C–F).

### 3.3. Effect of SFN on Oxidative Stress Markers

To determine the functional effect of Nrf2 activation via SFN following SE on oxidative stress markers, we measured the levels of oxidized and reduced forms of glutathione (GSSG and GSH, respectively) as well as their ratio (GSH: GSSG).

**Figure 3:**
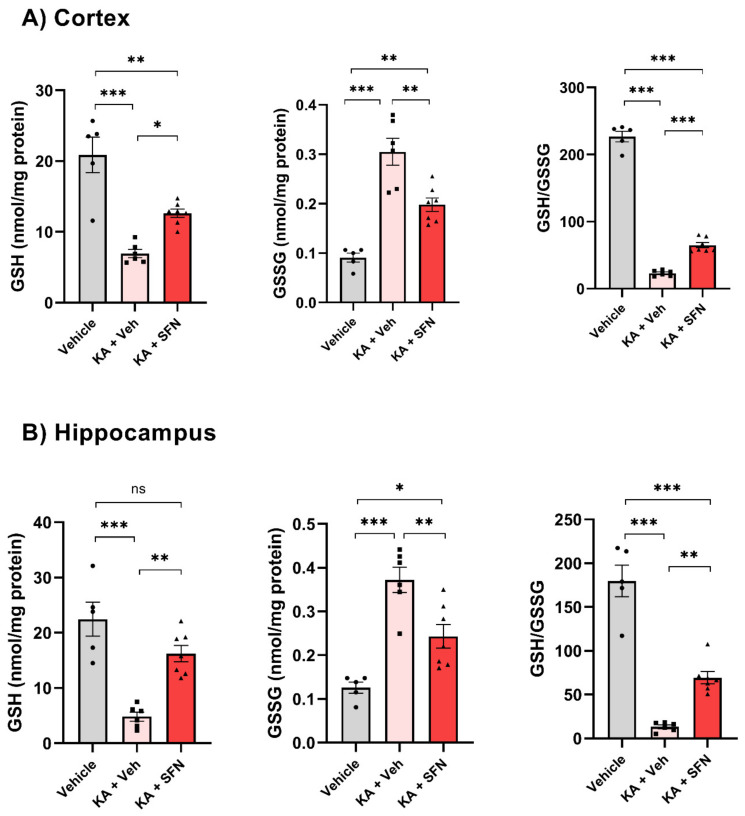
**SFN improves oxidative stress markers following in vivo SE.** Reduced (GSH) and oxidized (GSSG) glutathione levels, and their ratio (GSH/GSSG) in the cortex (**A**) and hippocampus (**B**) of sham-operated rats (treated with vehicle, *n* = 5) and rats subjected to KA-induced SE treated with either vehicle (*n* = 6) or SFN (5 mg/kg/day over 5 days, *n* = 7), and sacrificed 7 days after SE onset. Data were analysed by one-way ANOVA followed by Tukey’s multiple comparisons test. Bars represent the mean ± SEM, (* *p* < 0.05, ** *p* < 0.01, and *** *p* < 0.001).

Following KA-induced SE, the levels of GSH were significantly decreased in the cortex (~67%) and in the hippocampus (~79%) when compared to vehicle-treated animals (Figure 3A,B; A: F (2, 15) = 26.37, *p* < 0.0001; B: F (2, 15) = 22.36, *p* < 0.0001). Accumulation of GSSG, reduced form of glutathione, was significantly increased (approximately three-fold) in both the cortex (Figure 3A, F (2, 15) = 29.65, *p* < 0.0001) and hippocampus (Figure 3B, F (2, 15) = 21.02, *p* < 0.0001). In addition, a depletion of the ratio of GSH: GSSG was detected in both brain regions. Treatment with SFN (5 mg/kg/day over 5 days) significantly improves these oxidative stress markers.

In the SFN treated group, the GSH level has increased by two-fold in the cortex compared to the vehicle-treated group (6.9 vs. 12.6 nmol/mg protein; Figure 3A, *p* = 0.0145), and by three-fold in the hippocampus (4.8 vs. 16.2 nmol/mg protein; Figure 3B, *p* = 0.0010). SFN also decreased the GSSG levels by 35% in both cortex (Figure 3A, *p* = 0.0022) and hippocampus (Figure 3B, *p* = 0.0058). Accordingly, these changes in the levels of both glutathione forms resulted in an overall depletion of the ratio GSH/GSSG following SE in both cortex (Figure 3A, F (2, 15) = 457.1, *p* < 0.0001) and hippocampus (Figure 3B, F (2, 15) = 66.83, *p* < 0.0001). Treatment with SFN over 3 days following SE resulted in a three-fold increase in GSH/GSSG ratio in the cortex (Figure 3A, *p* < 0.0001) and a five-fold increase in the hippocampus (Figure 3B, *p* = 0.0021) as compared to animals subjected to SE and treated with vehicle only.

### 3.4. SFN Increases Levels of Total Antioxidant Capacity Following SE

We next determined the effect of SFN on the cumulative effect of all antioxidants present in the blood and brain tissue of rats following SE in vivo. For this purpose, we determined the Total Antioxidant Capacity (TAC), using a commercially available assay kit, in four groups of animals: Vehicle treated (*n* = 6) and SFN treated (5 mg/kg/day over 5 days, *n* = 6), both were not subjected to SE. Rats subjected to KA-SE were treated with either SFN (5 mg/kg/day over 5 days, *n* = 6) or vehicle (equal volume and number of injections as SFN group, *n* = 6). All animals were sacrificed 7 days after the KA-SE (or the first administration of vehicle or SFN).

**Figure 4:**
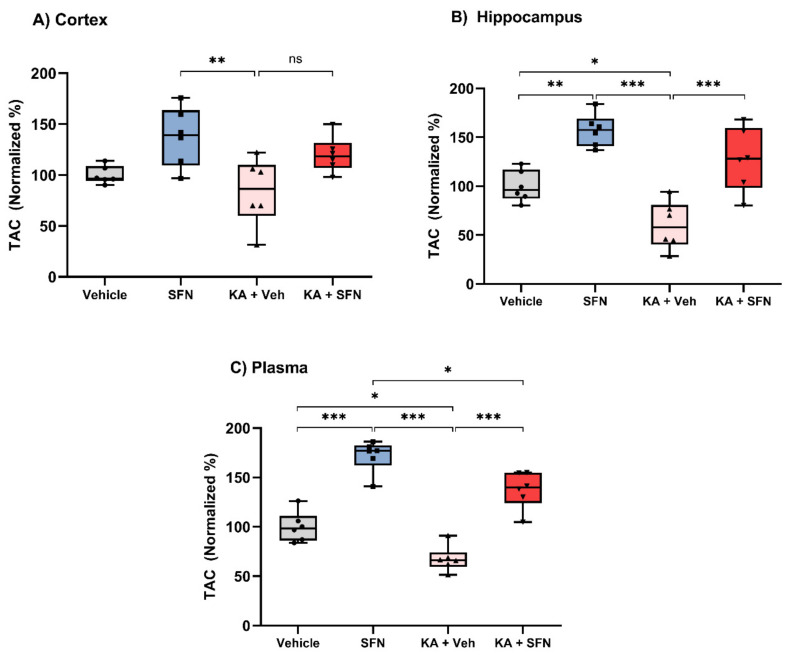
**SFN increases total antioxidant capacity following SE. Total antioxidant capacity normalized to Trolox standard in cortex (A), hippocampus (B) and plasma (C) of vehicle or SFN** (5 mg/kg/day for 5 days) treated rats (no SE), and rats subjected to KA-SE and treated with SFN (5 mg/kg/day for 5 days) or equivalent volume and number of vehicle injections. (**A**–**C**): *n* = 6. Data are expressed as the mean ± SEM, ((**A**): F (3, 20) = 5.673, *p* = 0.0056; (**B**): F (3, 20) = 18.68, *p* < 0.0001; (**C**): F (3, 20) = 48.56, *p* < 0.0001), one way-ANOVA followed by Tukey’s post hoc. * *p* < 0.05, ** *p* < 0.01, and *** *p* < 0.001 vs. KA + Vehicle group. n.s. *p* > 0.05.

Interestingly, in rats not subjected to SE and treated with SFN, the TAC levels were significantly increased, compared to the vehicle, in the hippocampus (Figure 4B, *p* = 0.0022) and plasma (Figure 4C, *p* < 0.0001). The TAC levels in the cortex of these animals were also increased; however, this increase did not reach significance (Figure 3A, *p* = 0.0620). The KA-SE resulted in a significant depletion in TAC in levels in the hippocampus (Figure 4B, *p* = 0.0359) and plasma (Figure 4C, *p* = 0.0104), as compared to the vehicle-treated group. Interestingly, the TAC levels in the cortex of these animals were not significantly different from vehicle-treated rats that were not subjected to SE (Figure 3A, *p* = 0.6510).

### 3.5. SFN Is Neuroprotective against KA-SE Induced Neuronal Cell Death

**Figure 5:**
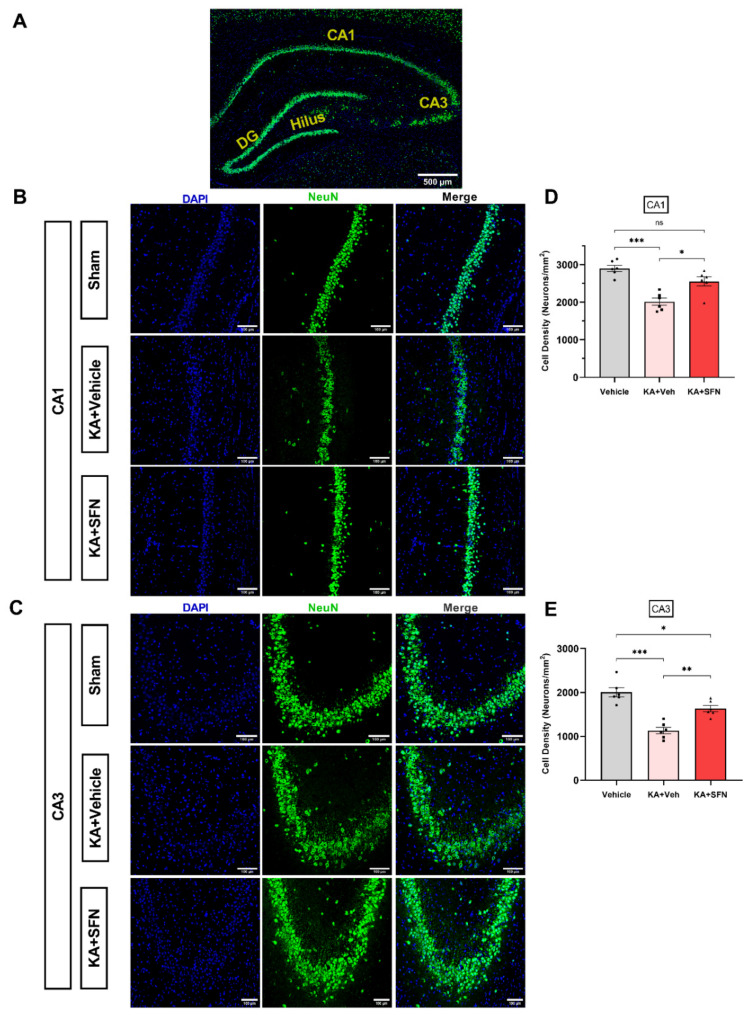
**SFN prevents neuronal cell loss in the hippocampus following SE in vivo.** (**A**). Analysed Regions of Interest (ROI) for CA1 and CA3 in the hippocampus. (**B**,**C**). Representative images of the CA1 and CA3 fields of the hippocampus of sham (vehicle-treated) controls (*n* = 6) and of rats treated with either SFN (5 mg/kg/day over 5 days, *n* = 6) or vehicle (equal volume and number of injections as SFN group, *n* = 6), 7 days following KA-SE onset. DAPI (Blue), and NeuN (Green) labelling, top to bottom. Scale = 500 µm in (**A**) and 100 µm in (**B**,**C**). (**D**,**E**). Cell densities in the CA1 and CA3 fields of animals in B and C, respectively. Data are expressed as the mean ± SEM. * *p*< 0.05, ** *p* <0.01, and *** *p* < 0.001 compared to sham group by one-way ANOVA followed by Tukey’s post hoc test. [(**D**): F (2, 15) = 19, *p* < 0.0001; (**E**): F (2, 15) = 26, *p* < 0.0001].

We asked whether the increase in GSH, the improved overall ratio of GSH/ GSSG and the increase in TAC following KA-SE in vivo is associated with neuroprotection. We measured the neuronal cell densities in CA1 and CA3 hippocampal subfields one week following KA-SE. In CA1, there was 30% of neuronal loss in the KA-SE vehicle-treated animals as compared to vehicle-operated rats (Figure 5B,D; *p* < 0.0001), while, in CA3, a greater loss (44%) in neuronal density was observed (Figure 5C,E; *p* < 0.0001).

This neuronal loss in both CA1 and CA3 was significantly ameliorated by SFN (Figure 5B–E). Interestingly, the neuronal density in CA1 of animals subjected to KA-SE and treated with SFN was not significantly different from that of sham animals (treated with vehicle, no SE; Figure 5D, *p* = 0.0723). In the CA3 subfield of SFN treated rats, the neuronal loss was decreased from 44% (in the vehicle group) to 19% in the SFN group.

## 4. Discussion

We report that KA-induced SE resulted in increased OS markers and neuronal cell death in the hippocampus and cortex and that prolonged treatment with the naturally occurring, phytochemical isothiocyanate Nrf2 activator SFN attenuated OS in the brain and plasma, increased the antioxidant capacity in the brain, and thus significantly decreased the SE-induced neuronal cell death.

OS is a complex and dynamic situation characterized by an imbalance between the production of ROS and the availability and action of antioxidants [29]. Endogenous antioxidants may be insufficient to protect the cell against the induced oxidative damage. Therefore, direct covalent inhibitors, like SFN, that interrupt the Keap1-Nrf2 protein-protein interaction (PPI) leading to Nrf2 activation have attracted great attention as potential preventive and therapeutic agents for OS-related diseases [30]. Animal models of acquired epilepsy provide evidence of profound changes in ROS production, antioxidant capacity, and neuronal viability as a result of various epileptogenic injuries. These alterations occur rapidly after the inciting event and persist during epileptogenesis [19,31].

Several previous studies have reported the potential neuroprotective effects of Nrf2 activation in epilepsy animal models [7,18,19]. We recently reported the potential antiepileptogenic, antiepileptic, and neuroprotective effects of cyclic cyanoenones Nrf2 activators alone [18] or in combination with NADPH oxidase inhibitor [7] following the KA-induced SE TLE model. In another study, we showed that SFN, together with an acute antioxidant drug N-acetylcysteine (NAC), reduced OS stress in the brain and blood during epileptogenesis in an electrical induced SE rat model of acquired epilepsy, and improved pathological outcomes by providing a blockade of spontaneous seizure progression and reduction of neuronal cell loss [19].

However, several limitations of these studies brought the therapeutic effects of SFN into question. First, SFN is non-selective, affecting many off-target proteins [20], and can itself exhibit both anticonvulsant [22] and proconvulsant activity [21]. Moreover, SFN is toxic at high doses and has probable poor penetration of the blood-brain barrier [32]. Finally, a convincing effect of SFN on epileptogenesis has only been shown when it was administered in combination with a high dose antioxidant, NAC [19].

To this end, we aimed in the present study to investigate the antioxidant and therapeutic effects of SFN in a chemoconvulsant TLE rat model. We first show, using an in vitro model of persistent seizure-like activity, that SFN can prevent ROS production, increase GSH levels, and consequently prevent seizure-like activity-induced neuronal death.

We then asked whether these antioxidant and neuroprotective properties can be translated in vivo. We first tested whether the systemic SFN administration can rescue Nrf2 and target genes in the cortex and hippocampus following KA-induced SE TLE model in rats. Indeed, we showed that Nrf2 levels were decreased following in vivo SE in both brain regions, and that treatment with SFN significantly increased the protein and mRNA levels of Nrf2, and its target genes NQO1 and HO-1. The increased levels of NQO1, a prototypic Nrf2 target enzyme [18] indicate that SFN increases not only the levels of Nrf2 but also its functional activity.

Further, we looked at the levels of reduced glutathione (GSH), the major endogenous antioxidant considered to be one of the most important scavengers of ROS in the central nervous system, the levels of oxidized glutathione (GSSG), and their ratio, which is used as a marker of OS and often also as a marker of cellular toxicity. KA-induced SE significantly decreased the GSH and increased the GSSG levels in both cortex and hippocampus. Accordingly, the GSH: GSSG ratio was dramatically decreased following the SE, while, following the treatment with SFN, this ratio was increased, however, below its baseline levels as compared to the sham operated group.

We also demonstrated other beneficial changes in OS markers regarding TAC. SFN treatment was associated with a significant increase of TAC in blood and hippocampus. In the cortex, there was also a trend of increase in TAC; however, this did not reach statistical significance. Importantly, SFN treatment in sham animals (not subjected to SE), also showed similar behaviour.

Finally, human data demonstrate numerous alterations in the hippocampus of TLE patients, including hippocampal sclerosis (HS) and cell death [33], and experimental models of TLE (mainly KA-induced SE) also showed high death rates in hippocampi, with the most noticeably changes appear in CA1 and CA3 hippocampal subregions [34,35,36,37]. We therefore asked whether the activation of Nrf2, and the related antioxidant effects showed above can be neuroprotective and decrease neuronal cell death in these two subregions.

Indeed, our results confirmed previous findings demonstrating a significant cell loss in the CA1 and CA3 hippocampal subregions of rats subjected to KA-induced SE, and show that treatment with SFN for 5 days after SE significantly decreased the neuronal cell loss in these regions.

In conclusion, the results reported here suggest that oxidative processes may contribute to SE-induced neurodegeneration and clearly indicate that antioxidant treatment via Nrf2 activation using SFN can exert neuroprotection against SE-induced toxicity and has potential as a disease-modifying treatment in epilepsy.

## 5. Conclusions

We presented data on the antioxidant properties and neuroprotective effects of SFN, a naturally occurring Nrf2 activator, as monotherapy following KA-induced SE in rats. This model recapitulates the histological and neurophysiological changes that have been observed in human TLE, where seizures originate from the hippocampus and drug resistance is a particular problem. Since many of these epilepsies are acquired conditions following an insult to the brain, such as a prolonged seizure and traumatic brain injury, and since oxidative stress has been observed in animal models of these conditions, as well as in epilepsy patients, targeting oxidative stress by Nrf2 activation (either as a monotherapy or as adjuvant therapy) may provide neuroprotective effects and modify the development of epilepsy and associated neurodegenerative diseases.

## Figures and Tables

**Table 1:** Primers used.

Name	Forward	Reverse	Ampli. Size
Nrf2	GCAACTCCAGAAGGAACAGG	GGAATGGCTCTCTGCCAAAAGC	203
GAPDH	GACATGCCGCCTGGAGAAAC	AGCCCAGGATGCCCTTTAGT	92
NQO1	GTTTGCCTGGCTTGCTTTCA	ACAGCCGTGGCAGAACTATC	99
HO-1	ACAGGGTGACAGAAGAGGCTAA	CTGTGAGGGACTCTGGTCTTTG	107

Nrf2 nuclear factor E2-related factor 2, NQO1 NAD(P)H:quinone oxidoreductase-1 gene, HO-1 haem oxygenase 1 gene.

## Data Availability

The data presented in this study are available in article.

## Supplementary Materials

The following are available online at https://www.mdpi.com/article/10.3390/antiox10111702/s1, Figure S1: Representative graph of synchronous Ca^2+^ signal in neurons following low Mg^2+^ aCSF. Figure S2: Representative photographs of co-staining of primary neuronal cultures with Hoechst 33342 and PI. Figure S3: Representative photographs of primary neuronal cultures stained with MCB dye.

## Abbreviations

aCSF	artificial cerebrospinal fluid
AEDs	anti-epileptic drugs
DHE	dihydroethidium
2HE	2-hydroxyethidium
GSH	reduced glutathione
GSSG	oxidized glutathione
HO-1	heme oxygenase 1
KA	Kainic Acid
KEAP1	Kelch ECH associating protein 1
MCB	Monochlorobimane
NQO1	NAD(P)H: quinone oxidoreductase 1
Nrf2	Nuclear factor erythroid 2-like 2
OS	oxidative stress
ROS	Reactive oxygen species
SE	Status epilepticus
SFN	Sulforaphane
TAC	Total antioxidant capacity
TLE	Temporal lobe epilepsy
