# Supplementary figures and images for: Induction of the Nrf2 Pathway by Sulforaphane Is Neuroprotective in a Rat Temporal Lobe Epilepsy Model
Source: Antioxidants (Basel). 2021 Oct 27;10(11):1702. doi: 10.3390/antiox10111702 (PMC8615008; doi:10.3390/antiox10111702)

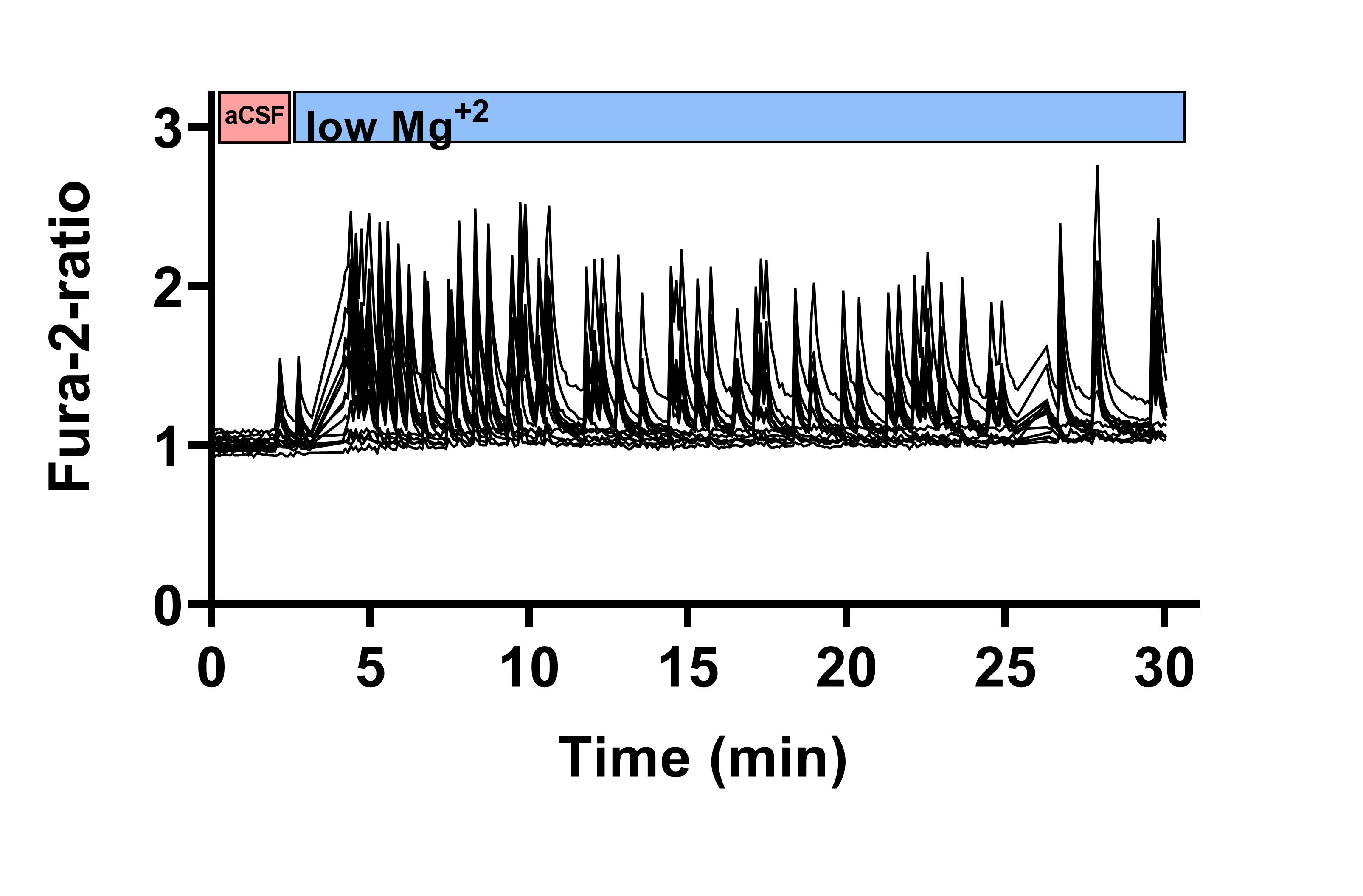

Supplement: Supplementary file 1 [file antioxidants-10-01702-s001.zip › antioxidants-1417828-supplementary Figure S1.tif]

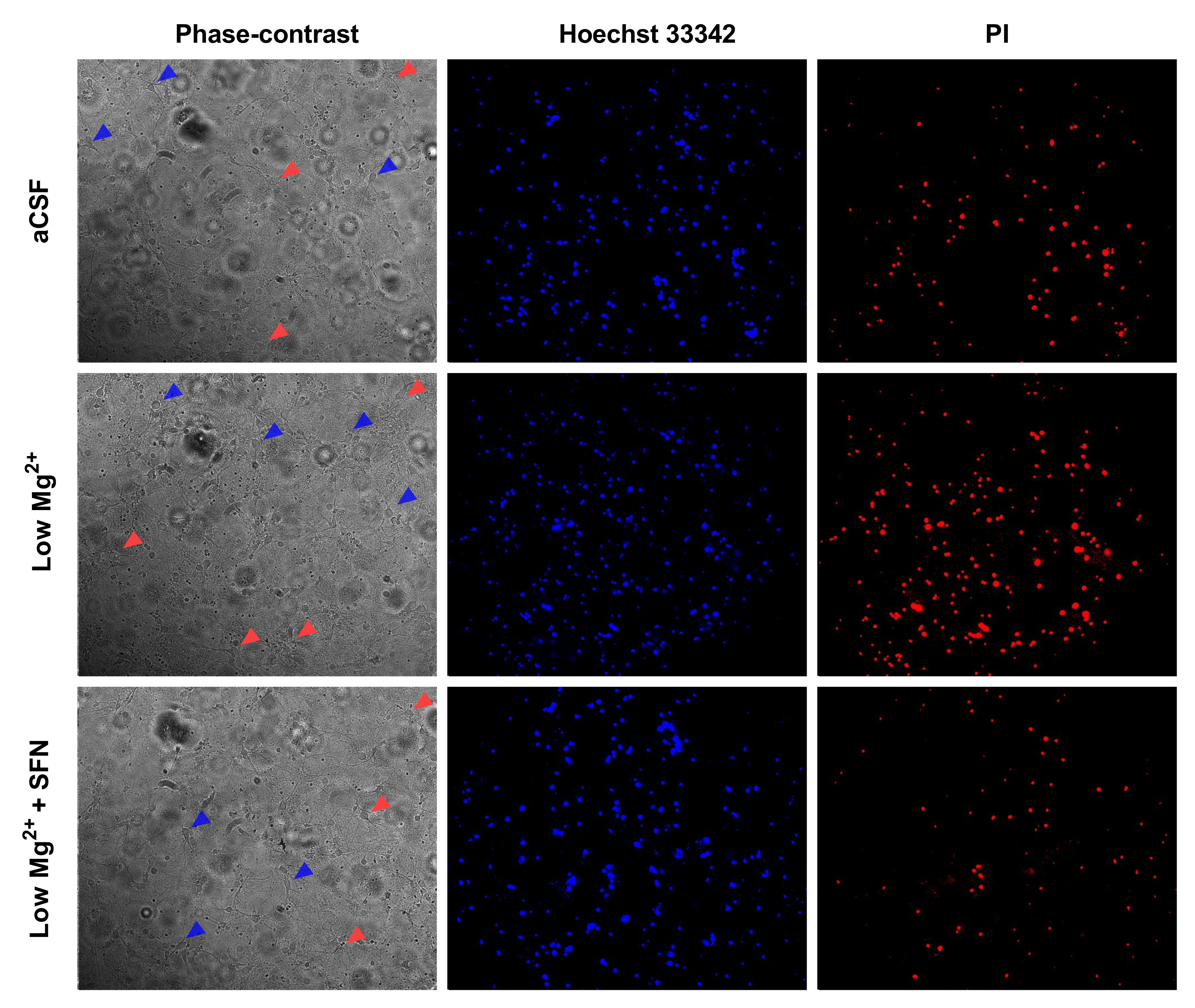

Supplement: Supplementary file 1 [file antioxidants-10-01702-s001.zip › antioxidants-1417828-supplementary Figure S2.tif]

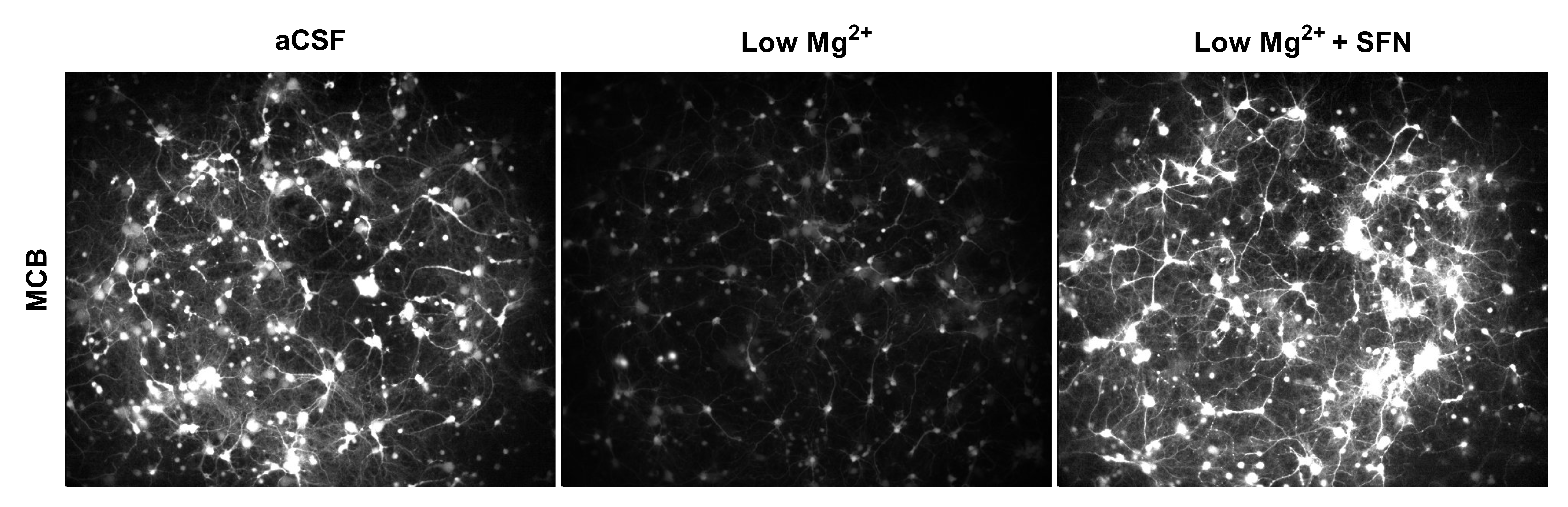

Supplement: Supplementary file 1 [file antioxidants-10-01702-s001.zip › antioxidants-1417828-supplementary Figure S3.tif]
